# Low level perceptual, not attentional, processes modulate distractor interference in high perceptual load displays: evidence from neglect/extinction

**DOI:** 10.3389/fpsyg.2013.00966

**Published:** 2014-01-10

**Authors:** Carmel Mevorach, Yehoshua Tsal, Glyn W. Humphreys

**Affiliations:** ^1^Behavioural Brain Sciences Centre, School of Psychology, University of BirminghamBirmingham, UK; ^2^Department of Psychology, Tel Aviv UniversityTel Aviv, Israel; ^3^Department of Psychology, The College of Management Academic StudiesIsrael; ^4^Department of Experimental Psychology, University of OxfordOxford, UK

**Keywords:** attention, perceptual load, dilution, neglect, extinction

## Abstract

According to perceptual load theory (Lavie, 2005) distractor interference is determined by the availability of attentional resources. If target processing does not exhaust resources (with low perceptual load) distractor processing will take place resulting in interference with a primary task; however, when target processing uses-up attentional capacity (with high perceptual load) interference can be avoided. An alternative account (Tsal and Benoni, 2010a) suggests that perceptual load effects can be based on distractor dilution by the mere presence of additional neutral items in high-load displays so that the effect is not driven by the amount of attention resources required for target processing. Here we tested whether patients with unilateral neglect or extinction would show dilution effects from neutral items in their contralesional (neglected/extinguished) field, even though these items do not impose increased perceptual load on the target and at the same time attract reduced attentional resources compared to stimuli in the ipsilesional field. Thus, such items do not affect the amount of attention resources available for distractor processing. We found that contralesional neutral elements can eliminate distractor interference as strongly as centrally presented ones in neglect/extinction patients, despite contralesional items being less well attended. The data are consistent with an account in terms of perceptual dilution of distracters rather than available resources for distractor processing. We conclude that distractor dilution can underlie the elimination of distractor interference in visual displays.

## INTRODUCTION

Everyday situations require a flexible and efficient selection mechanism that helps us deal with complex perceptual input, only a small portion of which is important for our current behavioral goal. Attentional selection is therefore required to facilitate the processing of targets and the inhibition (or filtering-out) of distractors. However, such selection mechanisms are not always optimal. Indeed, various scenarios may yield distractor processing that interferes with target responses (distractor interference).

Considerable research has been dedicated to describing the factors that influence such distractor interference (e.g., Eriksen and Eriksen, 1974; Lachter et al., 2004). In particular, perceptual load theory (Lavie and Tsal, 1994; Lavie, 1995) proposes that the capacity of attention resources is at the heart of distractor interference. That is, when target processing does not exhaust attentional capacity (when there is a low perceptual load), left-over resources will be allocated to the distractors, thereby producing interference. Conversely, when attentional resources are depleted (when there is a high perceptual load), distractor processing is reduced and consequently no distractor interference is observed. Thus, according to this account, the perceptual load present in a given task will determine the level of attention resources available and hence whether distractor interference occurs.

Studies looking into the effect of perceptual load on distractor interference have frequently contrasted conditions of low-load (where the target appeared by itself in one of several possible central positions) with conditions of high-load (where the target was embedded among several central neutral items; e.g., Bavelier et al., 2000; Lavie and Fox, 2000; Beck and Lavie, 2005). The efficacy of selection is measured by the effect of an incongruent relative to a neutral distractor appearing in the display. Typically, substantial interference is observed under low-load conditions, but this is either markedly reduced or completely eliminated under high-load conditions. According to perceptual load theory (e.g., Lavie, 1995), this reduced interference under the high-load condition is a direct consequence of depleted attentional resources which are required for processing the central display, leaving fewer spare resources to be captured by the irrelevant distractor. While the precise definition of perceptual load has been elusive a recent attempt (Torralbo and Beck, 2008) points to the amount of local competition for attention (potentially at a neuronal level) as the determinant of the perceptual load of the target and therefore the amount of resources that will be allocated for its processing (and consequently the remaining resources that will be allocated to distractor processing).

Recently, an alternative explanation has been proposed for the lack of distractor interference under conditions of high perceptual load. In a series of studies Tsal and Benoni (Benoni and Tsal, 2010, 2012; Tsal and Benoni, 2010a; Benoni et al., 2013) have proposed that low-level perceptual processes, rather than the availability of attentional resources, can explain reduced distractor interference. According to this account, the neutral items present in high-load displays (but not in low-load displays) compete together for perceptual representation. When distractors have similar features, their perceptual weight is jointly decreased (see Duncan and Humphreys, 1989) and as a consequence distractor representations are weakened and their effect on target identification diminishes (and distractor interference deceases). Critically, this alternative account holds that effects of perceptual dilution will occur even if the neutral elements are not attended, since their impact is at a pre-attentional, perceptual level of representation.

The dilution account has been supported in experiments using a variety of converging operations. For example, in one experiment Tsal and Benoni (2010a) presented the same multiple color display in a low-load (but high dilution) condition and in a high-load (and high dilution) condition (Tsal and Benoni, 2010a). However, in the former the target color was pre-known, thus allowing for a low-load processing mode, whereas in the latter it was not, hence necessitating an active search of the entire display. In another experiment, Tsal and Benoni (2010a) spatially separated the additional non-target items from the target item and thus introduced another condition in which perceptual load is low but dilution is high. Distractor interference was abolished under both conditions, giving support to the argument that the mere presence of non-target items rather than the perceptual load associated with target processing eliminates distractor interference. Similar evidence for the reduction of distractor interference with the addition of task-irrelevant elements – the dilution phenomenon – has been reported several other times in the literature (e.g., Kahneman and Chajczyk, 1983; Brown et al., 1995; Roberts and Besner, 2005). Note that, on this account, the manipulation of perceptual load where additional non-target items are introduced in the display for high-load conditions cannot distinguish between accounts highlighting the availability of attentional resources (perceptual load) or those highlighting low level perceptual processes (dilution) in determining distractor interference.

Recently, Lavie and Torralbo (2010) have argued that the dilution effect reported by Tsal and Benoni (2010a) is brought about by attentional factors that could still be incorporated within the perceptual load explanation. That is, it can be hypothesized that the non-target items presented in a dilution display attract attention in the same way that a distractor attracts attention in low-load displays. For instance, spatially separating the non-target items from the target will mean that only limited attentional resources are needed for target processing. The consequence of this may be that the remaining attentional resources can be allocated to both the distractor (as in the low-load displays) and the non-target items (as in the high-load displays). It follows that fewer resources are available for distractor processing compared with the low-load condition. This would result in reduced distractor interference. This proposal remains to be tested.

In order to contrast the two competing explanations of non-target items either attracting resources, or weakening perceptual representations of the distractor, we tested effects of dilution/load based on the addition of non-target items to a display, using patients with unilateral neglect/extinction. These patients are of interest because they are typically thought to allocate less attention to the contralesional side compared with the ipsilesional side of space (e.g., Heilman and Valenstein, 1979; Duncan et al., 1997). It should be noted that while somewhat different accounts have been proposed to explain unilateral neglect (e.g., a deficit in disengagement, Posner et al., 1984; hemispheric imbalance, Kinsbourne, 1987; to name two classical accounts) they do not challenge the premise that contralesional elements receive reduced attention resources (or none at all). It follows that the attentional resources allocated to non-target items should be weakened when they fall in the contralesional field of such patients. According to perceptual load theory, the ameliorating effect of extra non-target items separated from the target should be greater when those items fall outside (compared with inside) the contralesional field, since the items primarily consume attentional resources when they do not fall in the contralesional field. However when the items fall in the contralesional field they do not compete so strongly for attention leaving sufficient resources for other distractors to be processed and interference to occur. On the other hand, if additional items dilute the perceptual processing of other distractors, then the additional items may reduce distractor interference even when they fall on the contralesional side. We presented a target along with distractor stimuli (items that could be congruent or incongruent with the response to the target) in vertical arrays at the center of the screen (ensuring that the patients could respond to the stimuli). In the critical conditions we examined the effects of presenting neutral (non-response-related) stimuli in the contra- and ipsilesional fields of the patients. We assessed whether the lateralised non-target items disrupted distractor inference specifically when they appeared on the contralesional side – since this is the condition in which the perceptual load and dilution accounts make opposite predictions.

## MATERIALS AND METHODS

### PARTICIPANTS

Seven patients were tested. Four had unilateral damage centered on the inferior, posterior parietal cortex (three right, one left hemisphere). One patient had a silent lesion in her left occipital cortex in addition to right parietal damage (JB). One had suffered anoxia and had bilateral degeneration along with a lesion pronounced in the left posterior parietal cortex (MH). One had bilateral lesions to right frontal and left occipito-temporal cortex (AS). JB and the unilateral right parietal patients all presented with neglect and/or extinction on the left side; this was designated the contralesional side for these patients. The other patients presented with right-side neglect and/or extinction; this was designated the contralesional side for these individuals (see **Table 1** for clinical details of the patients). Prior to participating in the study the patients were clinically assessed for their neglect/extinction symptoms. The clinical measure of neglect was based on the Apples cancelation task from the BCoS battery (Humphreys et al., 2012) which tests for both egocentric (missing targets across the page) and allocentric neglect (making false positive responses to distractors with a missing contralesional section, irrespective of their position on the page; see Bickerton et al., 2011). The clinical test of extinction, also from the BCoS, involved the patients detecting finger wiggles by the experimenter using unilateral or bilateral stimulus presentation conditions. A patient was classed as having extinction if they were worse at reporting a contralesional item under bilateral relative to unilateral conditions (age-matched controls do not show any such deficit under the standard clinical testing conditions; Humphreys et al., 2012).

**Table 1:** Demographic and clinical details for the patients.

Patients	Sex	Age	Lesion site	Clinical deficit	Etiology
JB	F	73	Right parietal and left occipital	Left allocentric neglect, left extinction	Stroke
MP	M	65	Right parietal-frontal-temporal	Left egocentric neglect and left extinction	Stroke
RH	M	74	Left temporo-parietal	Right allocentric neglect, right extinction	Stroke
MH	M	56	Left parietal plus bilateral degeneration	Right extinction	Anoxia
MC	M	62	Right temporo-parietal	Left egocentric neglect, left extinction	Stroke
AS	M	65	Right frontal and left occipito-temporal	Right extinction	Stroke
RP	M	52	Right temporo-parietal	Left egocentric neglect, left extinction	Stroke

*The clinical measures here were based on the tests of spatial attention in the BCoS battery (Humphreys etal., 2012).*

In addition to this, the patients were given a lab test of neglect and extinction. In this case the patients were presented with unilateral or bilateral presentations of the letters A–D on a PC screen for 200 ms, with each letter appearing in either the left or right visual field. There were 24 unilateral left, 24 unilateral right, and 48 bilateral trials. A group of 20 normal participants, age-matched to the patients, made no more than one error when reporting the letters. The patients were classed as having extinction if they showed a drop in performance of 0.04 or more on bilateral relative to unilateral presentation trials (see Chechlacz et al., 2013). They were classified as showing some degree of neglect if they failed to report at least two items fewer on the contra- than the ipsilesional side under unilateral presentation conditions, and they passed the unilateral trials on the extinction test in the BCoS. All of the current patients either showed aspects of neglect or clinical extinction, relative to the age-matched control participants.

### STIMULI AND APPARATUS

A PC with a 19 in VGA color monitor was used to display the stimuli. The experiment was created and run with E-Prime software. The viewing distance was approximately 60 cm so that each cm on the screen represented 1° of visual angle. The stimuli were presented in black (for the target, distractor, and neutral letters) or blue (for the fixation dots) on a white background. The target letter was either x or z, presented in lower case (Ariel, 20 pts; 0.4° by 0.5° of visual angle in width and height, respectively). The distractor letters were uppercase X or Z, which could be congruent or incongruent with the response to the target (Ariel, Bold typeface, 24 pts; 0.67° by 0.8° of visual angle in width and height, respectively). There were five possible non-target letters: k, s, m, v, and n which appeared in lowercase (Ariel, 20 pts; 0.4° by 0.5° of visual angle in width and height, respectively).

The target letter was presented in one of four possible positions in a vertical column along the vertical meridian centered on the center of the screen and positioned 0.5° of visual angle apart (from edge to edge). The distractor letter was presented in one of two positions, either 1° of visual angle above or below the vertical column. We presented both targets and distractors centrally to prevent the patients’ lateralised attentional impairment affecting target or distractor processing. Instead of a single fixation point four blue dots presented in a central vertical column, 0.5 cm apart were used to direct the patients’ eyes to the center of the screen. These dots served as place holders for the possible positions of the target (were centered on the possible target letter position; i.e., could appear 0.75°, 1.75° of visual angle above and below the center of the screen).

**FIGURE 1:**
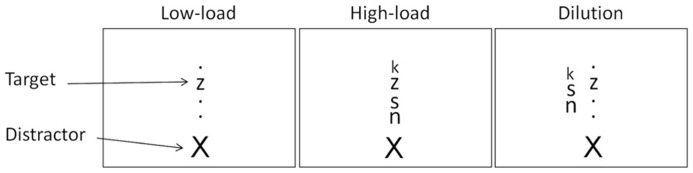
**Schematic view of the task.** In all of the conditions patients were asked to verbally report whether a target letter x or z appeared in the centrally presented target array. In the low-load condition the target letter appeared alone in the central target array. In the high-load condition the target was intermixed with three non-target letters and in the dilution condition the target appeared alone in the target array with the three non-target letters appearing to the left or the right of the target array. All conditions included the presentation of distractor above or below the target array. Patients were instructed to ignore the identity of the distractor.

Three types of display were used. In the low-load display only the target and distractor letters were presented, with the target appearing in one of the four possible locations and with the distractor appearing above or below the possible target locations. Three black dots were also presented in the remaining three possible target locations (i.e., the four possible target locations were filled with one target letter and three black dots, **Figure 1**). In the high-load display the target and distractor letters were presented in the same way as in the low-load display, with the only difference being the inclusion of three neutral letters presented in the three empty target positions. (i.e., the display included four letters in the central column, one of which was the target and three of which were non-target letters with the distractor appearing above or below the four letter array). Finally in the dilution display the target and distractor appeared in the same way as in the low-load condition, however, now the three neutral letters appeared on a separate vertical column falling 1° of visual angle to the left or right of the central column, with the middle letter positioned on the horizontal meridian and the two other letters 0.5° of visual angle above and below the central letter. The neutral letters in the dilution condition were presented to either the contralesional or ipsilesional side of space, this counterbalanced the trials and discouraged any spatial strategies the patients may use.

The identity of target, distractor and neutral letters were fully counterbalanced in each condition. Both the presentation of each of these letters, and the side on which the neutral letters appeared, were all equally frequent and randomly intermixed.

### PROCEDURE

The experimenter initiated each trial after verifying that the patient was ready and focused on the screen. Each trial began with the presentation of the fixation array for 1500 ms which was followed by a 500 ms blank interval, followed by the presentation of the target stimuli for 500 ms. The patients were required to make a verbal response regarding the identity of the target letter (x or z) with the experimenter then immediately pressing the corresponding key on the keyboard (“L” for z and “K” for x). We used this procedure as some of the patients found it challenging to maintain a stimulus-response mapping between symbolic stimuli and specific motor responses. The experiment included three types of 32-trial blocks (low-load, high-load, and dilution). Within each block half of the trials were congruent (i.e., target and distractor were both x or were both z) and half were incongruent (i.e., target x and distractor Z, or target z and distractor X). Each block was run twice so that eventually 64 trials were collected for each condition. Blocks were presented in random order and separated by rest periods. As explained above we were primarily interested in the dilution condition when the non-target letters were presented to the contralesional side for the patient. Thus, the dilution blocks were further split into contralesional dilution and ipsilesional dilution conditions.

Prior to the beginning of the experimental run, three practice blocks of 16 trials each were given (one block per condition). During the practice blocks visual feedback on the screen was presented along with verbal feedback by the experimenter.

## RESULTS

### LABORATORY TEST OF NEGLECT/EXTINCTION

**Table 2** presents the performance of the seven patients on the laboratory test of extinction. All but one patient (MH) showed neglect on this test, with poor report of a single target on the contralesional side of space (with errors ranging from 13 to 45%). In addition, all patients showed increased errors to contralesional targets when presented together with an ipsilesional target (range: 15–88% errors). These data verify that all patients exhibited neglect or extinction (and mostly both).

**Table 2:** Data on the laboratory test of neglect/extinction.

Patients	Single	Double
JP	0.13	0.21
MP	0.42	0.88
RH	0.29	0.63
MH	0	0.15
MC	0.42	0.63
AS	0.45	0.38
RP	0.17	0.63

*The table depicts the error rates exhibited by the patients when they had to identify an item presented to their contralesional side of space on its own (Single) or when the item co-occurred with another item presented to their ipsilesional side of space (Double). The report of ipsilesional items was in all cases at ceiling. A difference of 0.04 between the single and double item conditions indicates an extinction deficit that was outside of two SDs of the mean difference shown by a group of 20 age and education-matched control participants. The control participants also made no errors on single item trials. All the patients apart from MH showed a clinical deficit for single contralesional items, and all apart from AS showed a further drop in performance (extinction) for contralesional items on double item trials.*

### EFFECTS OF PERCEPTUAL LOAD/DILUTION

**FIGURE 2:**
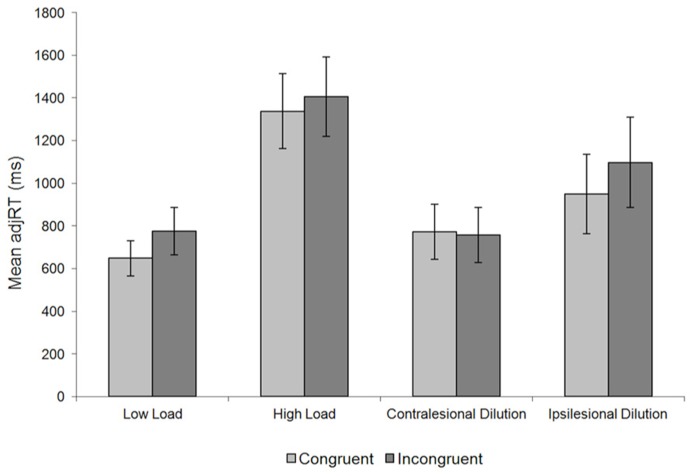
**Mean adjRT (±SEM) of patients’ performance in the load/dilution task**.

To incorporate both reaction times (RTs) and accuracy in a single measure and to avoid data contamination from a speed-accuracy trade off performance in the load/dilution task was assessed using an ANOVA on adjRT (RT/accuracy) with condition (low-load, high-load, contralesional dilution, and ipsilesional dilution) and congruency (congruent vs. incongruent) as within-subject factors. The adjRT data are depicted in **Figure 2**. A main effect of condition [*F*(3,18) = 11.507, *p* = 0.001, partial eta squared = 0.729] indicated that overall performance in the various load/dilution conditions differed.

Planned comparisons showed that adjRTs in the low-load (712 ms) condition were faster compared to the high-load condition [1372 ms; *t*(6) = 5.735, *p* = 0.001, Cohen *d* = 1.735] supporting the claim the two conditions differed in their perceptual load. Performance in the high-load condition (1372 ms) was also significantly slower than in the contralesional dilution condition [765 ms; *t*(6) = 9.398, *p* < 0.001, Cohen *d* = 1.47] but there was no overall difference in RTs between the low-load and contralesional dilution conditions [*t*(6) = 0.733, *p* = 0.491, Cohen *d* = 0.178]. This pattern verifies that both the low-load and the contralesional dilution conditions had similar levels of perceptual load which increased for the high-load condition. In addition, presenting the non-target letters in the ipsilesional side of space for the patients (in the ipsilesional dilution condition) significantly slowed-down performance (1024 ms) compared with the low-load condition [*t*(6) = 2.767, *p* = 0.033, Cohen *d* = 0.861]. This pattern is consistent with ipsilesional distractors attracting attentional resources away from the centrally presented targets (e.g., Ladavas, 1987).

The ANOVA further resulted in a significant two-way interaction of condition and congruency [*F*(3,18) = 4.239, *p* = 0.023, partial eta squared = 0.486]. This interaction is most important here as our investigation focuses on the conditions which modulate distractor interference, manifested as a difference in performance between congruent or incongruent displays that is assessed using planned comparisons. As in previous perceptual load experiments we found that congruent (648 ms) and incongruent (775 ms) displays differed significantly under the low-load condition [*t*(6) = 2.904, *p* = 0.027, Cohen *d* = 0.487] but not under high-load condition [1338 vs. 1405 ms for congruent and incongruent, respectively; *t*(6) = 1.405, *p* = 0.21, Cohen *d* = 0.14]. Thus, the low-load display yielded significant distractor interference while the high-load condition did not. Critically, we also found no distractor interference in the contralesional dilution condition where patients performed similarly in congruent (773 ms) and incongruent (757 ms) displays [*t*(6) = 0.71, *p* = 0.504, Cohen *d* = 0.047]. Thus, distractor interference was eliminated by introducing contralesional neutral letters. Finally, we also found significant distractor interference in the ipsilesional dilution condition where performance was better for congruent displays (949 ms) than incongruent ones [1098 ms; *t*(6) = 2.680, *p* = 0.044, Cohen *d* = 0.407]. These results are inconsistent with the predictions of load theory (e.g., Lavie, 1995) but alternatively support the dilution account (e.g., Tsal and Benoni, 2010a) in showing that the mere presence of contralesional items led to the elimination of distractor interference, despite these items receiving reduced attention in the present patients. The results with extra-ipsilesional items are also inconsistent with load theory, since these items should attract attention and reduce resources to distractors (Lavie and Robertson, 2001).

## DISCUSSION

The failure to filter out distractor stimuli (here manifested as distractor interference between congruent and incongruent trials) has been previously attributed to the availability of spare attentional capacity (Lavie, 2005). According to this account, when the perceptual load associated with processing the target is low (low-load conditions), unused attentional resources are allocated to distracters which in turn produces distractor interference. In contrast, when the perceptual load associated with processing the target is high (under high-load conditions) there are no attention resources left to process the distractor. Under these conditions, distractor interference is reduced. Recently, however, it has been suggested that distractor interference is modulated by low-level automatic perceptual processes that weaken its perceptual representation rather than the availability of attentional resources (Benoni and Tsal, 2010, 2012; Tsal and Benoni, 2010a,b). Specifically, multiple non-target elements lead to the dilution of perceptual processing for distractors, reducing their interference effects. We evaluated these accounts here by testing for effects of neutral (non-target) distractors in the contra- and ipsilesional fields of patients manifesting neglect and/or extinction on responses to central targets and distractors, based on the premise that contralesional distractors will receive less attention than stimuli presented at the center or in the ipsilesional field. We found several results of interest.

First, the patients were overall quicker in the low-load than the high-load condition. This verifies that perceptual load was manipulated successfully in these displays. Furthermore, overall adjRTs in the contralesional dilution condition (when neutral items appeared on the contralesional side) resembled those of the low-load condition, verifying that both conditions imposed only relatively low perceptual load and that few attentional resources were recruited by the contralesional distractors.

Second, distractor interference (the difference in performance between congruent and incongruent displays) was evident in the low-load but not in the high-load displays. This replicates prior studies on the effects of perceptual load (e.g., Bavelier et al., 2000; Lavie and Fox, 2000; Beck and Lavie, 2005). Critically, however, distractor interference was also eliminated in the contralesional dilution condition. Given that contralesional distractors should attract substantially reduced attentional resources here (evidenced both by the overall faster adjRTs to central targets in this condition and the patients’ performance on the neglect/extinction lab test) this result is striking. The finding contradicts the idea that distractor interference is affected solely by the availability of spare attentional resources that could be allocated to distractor processing (Lavie, 1995). However, the result is consistent with perceptual dilution account (e.g., Benoni and Tsal, 2010, 2012; Tsal and Benoni, 2010a; Dittrich and Stahl, 2011; Marciano and Yeshurun, 2011; Wilson et al., 2011). According to this account, the perceptual processing of multiple items is diluted and, as this occurs at a pre-attentive level, the effect remains even when the items appear in the contralesional side of our patients.

As well as this result, we also found that distractor interference effects remained when the neutral letters appeared on the ipsilesional side. Again this result is difficult to reconcile with the standard account of perceptual load. Ipsilesional distractors should attract attention, given the biased allocation of spatial attention in our patients (see also Shalev and Humphreys, 2000). This should lead to resources being allocated to the target to reduce this competition leaving fewer resources to generate distractor interference (similar to the high-load condition). On the other hand, we would also expect the ipsilesional items to dilute perceptual processing, so a reduced effect of distractor interference is also predicted by the dilution account. However, it is possible that perceptual dilution still operated through the neutral items presented in the ipsilesional field, but this was overridden by attention to the ipsilesional stimuli. There is evidence that neglect and extinction to stimuli on the contralesional side can reflect enhanced attention to stimuli on the ipsilesional side (e.g., Ladavas, 1987; Shalev and Humphreys, 2000). Enhanced attention, even to items subject to perceptual dilution, may lead to resources being allocated to them and reducing attentional resources available for targets (in terms of load theory, this would be equivalent to the effects of an increased cognitive load). The result is that distractor interference then increases. Rather than the account of perceptual load, which assumes that competition between targets and distractors leads to exclusive allocation of resources to targets, this proposal holds that target-distractor competition weakens resource allocation to targets and increases interference.

In a previous study, Lavie and Robertson (2001) tested the performance of two neglect patients in a perceptual load task. A single distractor was presented on the ipsilesional side of space while perceptual load was manipulated by adding a single non-target item adjacent to a central target. The results of this study showed reduced distractor interference with the addition of a single non-target item. The data were used to suggest that in neglect patients, the addition of a single central item can lead to the enhanced focus of attention on the target (and reduced attentional resources being allocated to distractors). Note however, that the inclusion of the additional non-target item may also have diluted the perceptual strength of the distractor. Thus this result does not distinguish the load and dilution accounts. In the present study, however, we have contrasted the two alternative explanations directly by presenting letters to the contralesional side of space for the patients to prevent spontaneous allocation of attention toward them. This way, we were able to show that low-level perceptual processes and not the allocation of attentional resources, modulate distractor interference. If the allocation of attentional resources is critical to reducing distractor interference, as proposed by load theory, we should have still observed distractor interference effect here when non-target elements fall on the relatively unattended side of (contralesional) space. The data contradict this.

One may argue that the perceptual load account could be broadened to propose that unconsciously perceived stimuli also impose a perceptual load. Thus the contralesional non-target items presented here may increase the perceptual load associated with target processing. This argument may have merit if it was evident that the unconscious presence of the contralesional items imposed a high-perceptual load on target processing, so that target processing would then require more attention resource. The data does not support this idea however, as the contralesional condition here demonstrated low-load performance in our neglect/extinction patients based on absolute RTs.

The question still remains as to why distractor interference occurs at all and what are the dominant factors influencing efficient selection. Various authors have proposed factors other than load as major determinants of efficient selection (e.g., Paquet and Craig, 1997; Fournier et al., 2002; Johnson et al., 2002; Murray and Jones, 2002; Chen, 2003; Theeuwes et al., 2004; Eltiti et al., 2005; Chen and Chan, 2007; Cosman and Vecera, 2012). For example, Paquet and Craig (1997) found that efficient selection strongly depends on target-distractor similarity and that distractor interference could occur under low-load conditions for near but not far distractors. Chen (2003) showed that increasing perceptual load did not facilitate selection when both the distracting and the target stimuli were part of the same object. Theeuwes et al. (2004) argued that high-load and low-load conditions differ in attentional set. In high-load conditions participants engage in focused attention suitable for a serial search whereas in low-load conditions they employ a distributed mode which is suitable for identifying a single target that can occur in one of several positions. In support of their claim they showed that intermixing high-load and low-load displays abolished the load effect. Theeuwes et al. (2004) proposed that advance knowledge of perceptual load level rather than perceptual load *per se*, modulates the processing of irrelevant distractors.

In another study particularly relevant here, Eltiti et al. (2005) argued that the major factor contributing to effective selection is the relative salience of the target and the distractor rather than perceptual load. The idea that the salience of the distractor (rather than the perceptual load associated with the target) is of critical consequence in driving interference also fits with our results. Essentially, the dilution account suggests that the perceptual weight of the distractor is affected by the inclusion of non-target letters in the display. In other words, the presence of the non-target items reduces the perceptual saliency of the distractor which in turn results with reduced distractor interference.

A paper published in the present issue (Chen and Cave, 2013) further suggests that the dilution effect could be modulated by variables such as the spread of focused attention, the category of the stimulus, and preknowledge of the target. Future studies would need to further address the nature of the various factors influencing the processing of neutral items which in turn modulate the processing of distractors.

## CONCLUSION

We have reported data showing that, in patients with visual neglect and extinction, contralesional, non-target letters reduced interference on centrally presented targets produced by central distractors. This occurred even though there was evidence that the contralesional items did not attract attentional resources (e.g., overall adjRTs did not differ from when only the central stimuli appeared). This finding contradicts an account of distractor interference in terms of perceptual load associated with the target but it does fit with the idea of perceptual dilution of the distractor from non-target items. On the other hand, with ipsilesional neutral items, distractor interference was maintained – again counter to load theory. We attribute this last result to ipsilesional stimuli capturing attention and removing resources from targets, over and above any effects of perceptual dilution.

## Conflict of Interest Statement

The authors declare that the research was conducted in the absence of any commercial or financial relationships that could be construed as a potential conflict of interest.
